# Thrombocytopenia in neonatal sepsis: Incidence, severity and risk factors

**DOI:** 10.1371/journal.pone.0185581

**Published:** 2017-10-04

**Authors:** Isabelle M. C. Ree, Suzanne F. Fustolo-Gunnink, Vincent Bekker, Karin J. Fijnvandraat, Sylke J. Steggerda, Enrico Lopriore

**Affiliations:** 1 Division of Neonatology, Department of Pediatrics, Leiden University Medical Center, Leiden, the Netherlands; 2 Department of Pediatric Hematology, Academic Medical Center, Amsterdam, The Netherlands; 3 Sanquin Blood Supply, Department of Clinical Transfusion Research, Leiden, The Netherlands; 4 Division of Hematology/Immunology, Department of Pediatrics, Leiden University Medical Center, Leiden, the Netherlands; 5 Sanquin Blood Supply, Department of Plasma Proteins, Amsterdam, the Netherlands; Centre Hospitalier Universitaire Vaudois, FRANCE

## Abstract

**Objectives:**

Thrombocytopenia is a frequent problem in neonatal sepsis and is among the most predictive, independent risk factors for sepsis-associated mortality. This study aims to clarify the occurrence, severity and duration of thrombocytopenia in neonatal sepsis.

**Study design:**

A cohort study was carried out among all neonates with proven culture positive sepsis that were admitted to a tertiary NICU between 2006 and 2015 (n = 460). The occurrence, severity and duration of thrombocytopenia were recorded, as well as major bleedings and potential risk factors for mortality in neonatal sepsis.

**Results:**

Sepsis was diagnosed in 460 of 6551 neonates (7%). Severe thrombocytopenia (platelets ≤50*10^9^/L) occurred in 20% (92/460) of septic neonates. The median time for platelets to rise >100*10^9^ was 6.0 days (interquartile range 4.0–7.0). On multivariate analysis, maternal hypertension, intravascular thrombosis and Gram negative (as opposed to Gram positive) sepsis were independently associated with thrombocytopenia in neonatal sepsis. In severe thrombocytopenia, 10% (9/92) suffered a severe IVH, compared to 5% (20/356) in neonates with platelets >50*109/L (p = 0.125). 10% (9/92) suffered a pulmonary hemorrhage, compared to 2% (9/368) in neonates with platelets >50*109/L (p = 0.001). On multivariate analysis, thrombocytopenia and Gram negative (as opposed to Gram positive) sepsis were independently associated with neonatal mortality.

**Conclusions:**

Thrombocytopenia is independently associated with maternal hypertension, intravascular thrombosis and Gram negative sepsis. Thrombocytopenia in neonatal sepsis increases the risk of mortality nearly four-fold, with another six-fold increase in mortality in case of Gram negative sepsis.

## Introduction

Thrombocytopenia, defined as a platelet count below 150*10^9^/L, is a frequent problem in neonatal intensive care units, complicating the clinical course in 22–35% of intensive care admissions [1–3]. Recently, there has been wide interest in thrombocytopenia and especially the correlation between platelet count and clinically significant bleeding. This correlation appears to be less clear than initially assumed, while platelet counts are still universally used in transfusion guidelines. Factors other than platelet count seem to be more important determinants of the bleeding risk in neonates [4], warranting more research in the field of thrombocytopenia. One of the major cause of thrombocytopenia in neonates is sepsis and thrombocytopenia may rapidly become very severe with the lowest platelet count reached within 24–48 hours after onset of infection [5].

The pathogenesis of thrombocytopenia in neonatal sepsis is not completely understood and may help to solve the puzzle concerning platelet count and subsequent bleeding. It has been suggested that in neonatal sepsis endothelial damage activates reticule-endothelial removal of platelets. Thrombocytopenia occurs as ultimately the rate of platelet production falls behind platelet consumption, with a causative role for serum thrombopoietin levels [6–9].

The importance of the relationship between thrombocytopenia and sepsis was emphasized by identifying thrombocytopenia as one of the most predictive, independent risk factors for sepsis-associated mortality in very low-birth weight neonates [10]. Similar findings were found in adults with sepsis admitted to an intensive care unit, as patients with platelet counts <100*10^9^/L were more severely ill, had more shock and organ failure and had an increased mortality up to 1 year after intensive care admission [11]. This deserves further clarification of the diagnostic and prognostic meaning of thrombocytopenia in sepsis. This study aims to clarify neonatal sepsis and thrombocytopenia in terms of severity, clinical course and outcome of thrombocytopenia.

Sepsis should not be regarded as a homogenous entity, as it neglects the pathogenic and clinical differences between the various causative micro-organisms and clinical syndromes and presentations of neonatal sepsis. In this study we therefore choose to present the characteristics of sepsis-related thrombocytopenia separately for Gram positive and Gram negative bacteria. Previous studies have found either no convincing difference between the occurrence and course of thrombocytopenia in sepsis caused by either Gram positive or Gram negative bacteria [12], or have reported a higher incidence of thrombocytopenia in Gram negative sepsis [13–16]. Interpretation of these results is complicated by the wide range of gestational age of the populations studied and the focus of most studies on (extremely) preterm neonates.

The aim of this study is to clarify the occurrence, severity and duration of thrombocytopenia in a large cohort of neonates with proven sepsis and to analyze risk factors for mortality in neonatal sepsis.

## Methods

### Study population

This cohort study was carried out among all neonates with proven sepsis admitted to the neonatal intensive care unit (NICU) of the Leiden University Medical Centre (LUMC) from January 2006 to September 2015. The LUMC is a tertiary referral center in the Netherlands. Proven sepsis was defined as an episode of clinical illness as assessed by the attending physician, accompanied by a positive blood culture for either Gram negative or Gram positive bacteria [17].

To compare the clinical and hematological outcomes of Gram positive and Gram negative sepsis, neonates that had positive cultures for both types of bacteria were excluded. In neonates with multiple separate sepsis episodes, only the first sepsis episode was recorded. Neonates that were transferred within the first 24 hours after the clinical sepsis onset were also excluded, as well as neonates with other causes of neonatal thrombocytopenia such as fetal/neonatal alloimmune thrombocytopenia (FNAITP) or maternal immune thrombocytopenic purpura (ITP), exchange transfusion and cases of suspected contamination of blood culture (defined as no rise in CRP >10mg/L during sepsis episode and withdrawal of antibiotic treatment within 72 hours). In case of platelet aggregation in the EDTA tubes, the aggregated platelet count is usually shortly followed by a new count after notification by our lab.

### Data collection

The primary outcome of the study was the occurrence of thrombocytopenia in neonatal sepsis. Secondary outcomes were severity and duration of thrombocytopenia, as well as the occurrence of severe hemorrhage and mortality.

Data were extracted from the hospital’s patient database, including medical files and laboratory outcomes. All collected data were anonymized. The protocol for the research project has been approved by the Ethics Committee of our institution and conforms to the provisions of the Declaration of Helsinki.

The following maternal data were recorded: maternal hypertension and mode of delivery. Maternal hypertension was defined as a diastolic blood pressure of 90 mmHg or more on two occasions more than four hours apart, or a single diastolic blood pressure above 110 mmHg. The following neonatal data were recorded: gestational age, birth weight, small for gestational age (SGA, defined as a birth weight below the 10^th^ centile for gestational age [18]), very low birth weight (VLBW, defined as a birth weight below 1500 gram), gender, day of onset and duration of thrombocytopenia, occurrence of severe hemorrhage, use of platelet transfusions, occurrence of intravascular thrombosis (defined as the detection of a vascular thrombosis on ultrasound examination), length of hospital stay and neonatal mortality. The following hematologic parameters were recorded for each neonate: initial platelet count at (clinical) sepsis onset and lowest platelet count during sepsis episode.

Thrombocytopenia was defined as a platelet count below 150*10^9^/L and was classified as mild (101–149*10^9^/L), moderate (51–100*10^9^/L), severe (21–50*10^9^/L) or very severe (≤20*10^9^/L). Transfusion guidelines of our department state that a platelet transfusion is always given to neonates with a platelet count <30*10^9^/L (January 2007-November 2009) or <20*10^9^/L (since 2009, after a change in the national transfusion protocol [19]). In case of manifest bleeding or need of procedure with a risk of bleeding, and for neonates with a birth weight <1500 gram and gestational age <32 week that are clinically ill, the threshold for transfusion is 50*10^9^/L. Standard dose for platelet transfusions is 20*10^9^ cells/kg.

Severe hemorrhage was defined as the presence of severe intraventricular hemorrhage (IVH), grade 3 or 4 according to Papile, or severe pulmonary or gastrointestinal hemorrhage requiring treatment with platelet transfusions, volume expanders and/or red blood cell transfusions [20, 21].

Platelet counts before clinical sepsis onset (‘initial platelet count’) were recorded for each neonate and compared. Absolute platelet count decrease (initial platelet count minus lowest platelet count) was calculated to adjust for the confounding of the occurrence of (mild) thrombocytopenia due to other causes (such as growth restriction) at sepsis onset.

### Statistical analysis

We calculated that group sizes of at least 35 infants were required to demonstrate a 25% difference in occurrence of thrombocytopenia in Gram negative sepsis compared to Gram positive sepsis, with 0.05 significance and a power of 80% by two-tailed analysis. Results of normally distributed variables were compared between the Gram positive and Gram negative sepsis groups using the Student’s *t*-test for numerical variables and the χ^2^ test or Fisher’s exact test for categorical variables. The Mann-Whitney *U* test was used as non-parametric test for numerical variables that did not follow a normal distribution. A log rank test was run to determine if there were differences in the time for platelets to rise to >100*10^9^/L in thrombocytopenic neonates in Gram positive sepsis compared to Gram negative sepsis.

The following variables were studied in a univariate logistic regression model: SGA, mode of delivery, maternal hypertension, gestational age at birth, occurrence of intravascular thrombosis, thrombocytopenia <150*10^9^/L and Gram negative sepsis. Known significant predictors for mortality in the univariate analyses were included in a multivariate logistic regression model to adjust for potential confounding. Results are presented as odds ratios (OR) with 95% confidence intervals (95% CI). A *p*-value below 0.05 was regarded as statistically significant. Statistical analysis was performed using IBM SPSS Statistics 23.0 (Chicago, Illinois, USA).

## Results

The total number of NICU admissions during the study period was 6551. A total of 460 (7%) neonates fulfilled our eligibility criteria for proven sepsis and were included in the study. Gram negative bacteria were found in 48 (10%) neonates with sepsis, compared to 412 (90%) neonates with Gram positive sepsis.

We excluded 91 neonates with culture proven sepsis either because of suspicion of contamination (n = 38, 36 with Gram positive blood culture for coagulase-negative staphylococcus, two with Gram negative blood culture for *Escherichia coli* and *Haemophilus influenzae*), missing data on blood culture and/or hematological data (n = 21), transfer within 24 hours after clinical sepsis onset (n = 13), neonates with both a Gram negative as well as a Gram positive blood culture (n = 12), neonates who received one or more exchange transfusions (n = 4), maternal ITP (n = 2) and FNAITP (n = 1).

Among the neonates with Gram negative sepsis, *Escherichia coli* (21/48) was the most commonly cultured bacteria, followed by Enterobacter species (8/48) and Klebsiella (6/48). In the Gram positive sepsis group, in the vast majority of neonates a coagulase-negative staphylococcus (311/412) was cultured, whereas *Staphylococcus aureus* (60/412) and Group B streptococcus (32/412) were the other most common bacteria in this group.

Baseline characteristics of the cohort are presented in Table 1. Birth weight appeared to be lower in infants developing Gram positive sepsis, as well as the proportion of infants with VLBW (<1500 gram). Initial platelet count before clinical onset of sepsis was 218*10^9^/L (IQR 162.0–285.8) thrombocytes in Gram negative sepsis compared to 215*10^9^/L (IQR 168.3–280.5) thrombocytes in Gram positive sepsis (*p* = 0.867). The occurrence of an initial low platelet count below 150*10^9^/L was comparable in both groups, 17% (8/48) in Gram negative sepsis and 12% (49/412) in Gram positive sepsis (*p* = 0.336).

**Table 1:** Baseline characteristics of Gram positive and Gram negative sepsis groups.

	Gram positive (n = 412)	Gram negative (n = 48)	*p*-value
Female—n (%)	180 (44)	25 (52)	0.268
Caesarean delivery—n (%)	213 (52)	19 (40)	0.112
Maternal hypertension—n (%)	94 (23)	9 (19)	0.523
Gestational age at birth (weeks)—median (IQR)	29.0 (27.0–31.0)	30.0 (28.0–33.0)	0.080
Birth weight (grams)—median (IQR)	1138 (913–1549)	1459 (1023–2139)	0.022
Small for gestational age (SGA)—n (%)	59 (14)	2 (4)	0.050
Very low birth weight (VLBW)—n (%)	299 (73)	27 (56)	0.018
Duration of hospitalization (days)—median (IQR)	23.0 (13.0–36.0)	16.0 (8.3–39.3)	0.069
Initial platelet count (*10^9^/L)—median (IQR)	215.0 (168.3–280.5)	218.0 (162.0–285.8)	0.867
Initial platelet count below 150*10^9^/L—n (%)	49 (12)	8 (17)	0.336

### Sepsis with and without thrombocytopenia

The differences between infants with sepsis with and without thrombocytopenia are presented in Table 2. Infants with sepsis that developed thrombocytopenia had a higher occurrence of caesarean delivery, maternal hypertension, vascular thrombosis and Gram negative sepsis. They were born with a lower birth weight and were more often born SGA.

**Table 2:** Differences between infants with sepsis with and without thrombocytopenia.

	Sepsis and thrombocytopenia (n = 226)	Sepsis without thrombocytopenia (n = 234)	*p*-value
Caesarean delivery—n (%)	125 (55)	107 (46)	0.040
Maternal hypertension—n (%)	64 (28)	39 (17)	0.003
Gestational age at birth (weeks)—median (IQR)	29.0 (27.0–31.0)	29.0 (27.0–31.0)	0.322
Birth weight (grams)—median (IQR)	1085 (878–1526)	1218 (962–1651)	0.021
Small for gestational age (SGA)—n (%)	38 (17)	23 (10)	0.027
Vascular thrombosis—n (%)	20 (9)	4 (2)	0.001
Gram negative sepsis^a^—n (%)	33 (15)	15 (6)	0.004
Duration of hospitalization (days)—median (IQR)	22.0 (12.0–37.0)	22.0 (12.0–36.3)	0.644

^a^As opposed to Gram positive sepsis.

On multivariate analysis, three variables were independently associated with the occurrence of thrombocytopenia in neonates with a septic event during NICU admission: maternal hypertension (OR 1.76, 95% CI 1.09–2.85, *p* = 0.021), intravascular thrombosis (OR 4.98, 95% CI 1.64–15.12, *p* = 0.005) and Gram negative (as opposed to Gram positive) sepsis (OR 2.74, 95% CI 1.42–5.30, *p* = 0.003).

### Characteristics of thrombocytopenia in neonatal sepsis

Thrombocytopenia occurred in 49% (226/460) of septic neonates. Severe and very severe thrombocytopenia (≤50*10^9^/L) was found in 20% (92/460) of neonates, 42% (20/48) of neonates with Gram negative sepsis, compared to 17% (72/412) of neonates with Gram positive sepsis (*p*<0.001). Platelet counts seem to fall lower in Gram negative sepsis, with a lowest median platelet count of 28*10^9^/L (Interquartile range (IQR) 13.0–85.5) and 66*10^9^/L (IQR 31.8–110.3) in Gram positive sepsis (*p* = 0.002). No difference was detected in time of onset of thrombocytopenia after the onset of sepsis, which was 1.0 day (IQR 1.0–2.0) in Gram positive sepsis and 1.0 day (IQR 1.0–1.0) in Gram negative sepsis (*p* = 0.157). In neonates with a lowest platelet count ≤100*10^9^/L, the median time for platelets to rise to >100*10^9^/L was 10 days in Gram negative sepsis (IQR 4.7–15.3) and 5 days in Gram positive sepsis (IQR 4.5–5.5, *p* = 0.002). 19% (86/460) neonates required a platelet transfusion. Significantly more neonates with Gram negative sepsis received a platelet transfusion, 40% (19/48) compared to 16% (67/412) of neonates with Gram positive sepsis (*p*<0.001). Within the subgroup of neonates requiring platelet transfusions, the median number of transfusions in Gram negative sepsis was 3 (IQR 1.0–7.0), versus 2 (IQR 1.0–3.0) in Gram positive sepsis (*p* = 0.009), Table 3. In the entire cohort, three neonates received 10 platelet transfusions or more, all three had proven Gram negative sepsis (*E*.*coli* n = 2, Serratia n = 1).

**Table 3:** Characteristics of thrombocytopenia in Gram positive and Gram negative sepsis.

	Overall (n = 460)	Gram positive (n = 412)	Gram negative (n = 48)	*p*-value^a^
Thrombocytopenia (<150*10^9^/L)—n (%)	226 (49)	193 (47)	33 (69)	0.004
Severity of thrombocytopenia				
- Mild (101–149*10^*9*.^*/L)—n (%)*	64 (14)	57 (14)	7 (15)	
- Moderate (51–100*10^*9*^*/L)—n (%)*	70 (15)	64 (16)	6 (13)	
- Severe (21–50*10^*9*^*/L)—n (%)*	58 (13)	54 (13)	4 (8)	
- Very severe (≤20*10^*9*^*/L)—n (%)*	34 (7)	18 (4)	16 (33)	
Days until platelet count >100*10^9^/L (days)^b^—median (IQR)	6.0 (4.0–7.0)	5.0 (4.0–7.0)	7.0 (5.3–12.8)	0.009
Lowest platelet count (*10^9^/L)—median (IQR)	63.0 (28.0–108.0)	66.0 (31.8–110.3)	28.0 (13.0–85.5)	0.002
Platelet count decrease (*10^9^/L)—median (IQR)	111.5 (58.8–164.3)	104.0 (55.0–151.0)	153.0 (68.8–222.5)	0.012
Day of onset after infection—median (IQR)	1.0 (1.0–1.0)	1.0 (1.0–2.0)	1.0 (1.0–1.0)	0.157
Proportion of neonates receiving platelet transfusion—n (%)	86 (19)	67 (16)	19 (40)	<0.001
Number of platelet transfusions per neonate^c^— median (IQR)	2.0 (1.0–3.0)	2.0 (1.0–3.0)	3.0 (1.0–7.0)	0.009

^a^ *p*-value of comparison between Gram positive and Gram negative sepsis.

^b^Recorded in neonates with lowest platelet count <100*10^9^/L, n = 137 in Gram positive sepsis and n = 26 in Gram negative sepsis.

^c^Recorded in neonates requiring platelet transfusions.

### Clinical outcome

Cranial ultrasound examination was performed in all but 13 cases (2 cases of Gram negative sepsis and 11 cases of Gram positive sepsis). 8 cases of severe IVH were excluded from analysis as these occurred before the presence of thrombocytopenia. Of the 92 neonates with severe and very severe thrombocytopenia (platelets ≤50*10^9^/L), 10% (9/92) had a severe IVH, compared to 5% (20/368) in neonates with platelets >50*10^9^/L (*p* = 0.125). Of the 29 neonates with severe IVH, 9 neonates (31%) had platelet counts below 50x10^9^/L, four neonates (14%) had moderate thrombocytopenia, two neonates (7%) had mild thrombocytopenia and 14 (48%) showed normal platelet counts. In our cohort no major gastrointestinal bleedings were reported. Pulmonary hemorrhages occurred in 4% (18/460) of septic neonates. Of the 92 neonates with platelets ≤50*10^9^/L, 10% (9/92) suffered a pulmonary hemorrhage compared to 2% (9/368) in neonates with platelets >50*10^9^/L (*p* = 0.001). Combined, a major bleeding defined as either a pulmonary hemorrhage or severe IVH occurred in 15% (14/92) of the 92 neonates with platelets ≤50*10^9^/L (4 neonates had both types of bleeding), compared to 8% (29/368) in neonates with platelets >50*10^9^/L (*p* = 0.031).

Overall mortality in our cohort was 5% (24/460) and was higher in neonates with platelets ≤50*10^9^/L (12%, 11/92), compared to neonates with platelets >50*10^9^/L (4%, 13/368) (*p* = 0.001). On multivariate analysis, two variables were independently associated with mortality in neonates with a septic event during NICU admission: thrombocytopenia <150*10^9^ (OR 3.77, 95% CI 1.33–10.64, *p* = 0.012) and Gram negative (as opposed to Gram positive) sepsis (OR 6.01, 95% CI 2.39–15.47 *p* = <0.001), Table 4.

**Table 4:** Multivariate analysis of predictors of mortality in neonatal sepsis.

	Deceased neonates (n = 24)	Surviving neonates (n = 436)	Univariate OR (95% CI)	*p*-value	Coefficient	Multivariate OR (95% CI)	*p*-value
SGA—n (%)	2 (8)	59 (14)	0.58 (0.13–2.54)	0.465	0.20	0.82 (0.17–3.96)	0.802
Caesarean delivery—n (%)	10 (42)	222 (51)	0.69 (0.30–1.58)	0.378	0.19	0.83 (0.32–2.16)	0.697
Maternal hypertension—n (%)	4 (17)	99 (23)	0.68 (0.23–2.04)	0.490	0.39	0.68 (0.20–2.27)	0.531
Gestational age ≤32 weeks—n (%)	20 (83)	347 (80)	1.28 (0.43–3.85)	0.656	0.53	1.70 (0.51–5.66)	0.386
Thrombocytopenia <150*10^9^—n(%)	19 (79)	207 (48)	4.20 (1.54–11.46)	0.003	1.33	3.77 (1.33–10.64)	0.012
Intravascular thrombosis—n (%)	1 (4)	23 (5)	0.78 (0.10–6.04)	0.812	0.78	0.46 (0.05–3.89)	0.475
Gram negative sepsis^a^—n (%)	10 (42)	38 (9)	7.48 (3.11–17.98)	<0.001	1.81	6.01 (2.39–15.47)	<0.001

^a^As opposed to Gram positive sepsis.

## Discussion

Thrombocytopenia is a common problem in neonates with proven sepsis, occurring in half of the cases with proven sepsis in our cohort. 20% of our cohort presented with very severe to severe thrombocytopenia (platelets ≤50*10^9^/L) and it takes a median 6 days for platelets to rise above 100*10^9^/L after the onset of sepsis. Our data show an almost 4-fold increase in mortality in septic neonates with thrombocytopenia. Additionally, neonates with Gram negative sepsis compared to Gram positive sepsis have a 6-fold risk of mortality.

In our study there is a significant baseline difference between birth weight and the proportion of VLBW infants in Gram positive compared to Gram negative sepsis. We assumed this to be due to the underlying distribution of bacteria, as the majority of our cohort had a positive blood culture for coagulase-negative staphylococci. These bacteria are highly associated with central venous catheterization and neonates with a lower birth weight (and lower gestational age) more often receive a central venous catheterization.

When comparing infants with sepsis with and without thrombocytopenia, there is a significant difference between the proportion of caesarean deliveries, maternal hypertension, birth weight, SGA infants, the occurrence of vascular thrombosis and Gram negative sepsis. The higher proportion of caesarean deliveries in infants with sepsis and thrombocytopenia is probably due to the higher occurrence of maternal hypertension and SGA infants in this group. Maternal hypertension is a known risk factor for neonatal thrombocytopenia, often an indication for a caesarean delivery and can cause intrauterine growth retardation. The exact pathogenesis of neonatal thrombocytopenia in maternal hypertension is unknown, but it is thought that maternal hypertension leads to fetal hypoxia, which has a depressant effect on fetal megakaryocytopoiesis and platelet production [22]. Intravascular thrombosis is highly associated with the use of vascular catheters. Catheters may cause increased platelet consumption by (1) causing mechanical damage to the vascular wall and alteration of the blood flow, but may also (2) consist of potentially thrombogenic material or may (3) be a medium to infuse damaging agents to the vascular walls [23]. Maternal hypertension, intravascular thrombosis and Gram negative (as opposed to Gram positive) sepsis were also independent risk factors for thrombocytopenia in multivariate analysis.

Although this study was not powered for outcome measurements of thrombocytopenia, a trend was shown towards a higher occurrence of severe IVH and a significant increase in the occurrence of pulmonary hemorrhage and in mortality in septic neonates with severe and very severe thrombocytopenia, compared to neonates with platelets >50*10^9^/L. On multivariate analysis, thrombocytopenia and Gram negative (as opposed to Gram positive) sepsis were independently associated with mortality in neonates with a septic event during NICU admission.

The clinical meaning of the higher occurrence of these bleeding complications and of mortality is unclear. The association and possible correlation between platelet count and clinically significant bleeding is not clear and remains controversial [4, 22, 23]. An incidence of 15% of major bleeding in severe thrombocytopenia in our cohort is in line with previous numbers reported in NICUs and underlines the complicated nature of the relationship between low platelet counts and severe bleeding events, as apparently a low platelet count is no clear indicator for the occurrence of severe bleeding events [23].

Previous studies show conflicting evidence on the association between the type of Gram stain and the occurrence of thrombocytopenia in neonatal sepsis. Manzoni et al. [11] did not find a difference in occurrence of thrombocytopenia comparing infections with Gram negative and Gram positive bacteria among very low-birth weight neonates, but used a different threshold for thrombocytopenia. In contrast, others found results similar to our findings and report a higher occurrence of thrombocytopenia in Gram negative sepsis [13–15]. However, sample sizes were generally small and results were obtained mostly from populations of very low-birth weight neonates and (very) preterm populations. Our results are obtained from a large, recent sample of neonates with sepsis and a wide range of gestational ages.

The pathogenesis of thrombocytopenia in neonatal sepsis is not clear. Thrombocytopenia may just be a marker of severity of sepsis, as Gram negative sepsis are more severe than Gram positive ones and sepsis can cause disseminated intravascular coagulation. A direct pathophysiological mechanism of endotoxins produced by Gram negative bacteria in neonatal sepsis could also contribute. Both bacterial groups show a great inhomogeneity, with a different pathogenicity for each separate bacterial species [24]. Theoretically, the observed differences in this study could be completely attributed to the individual pathogenic effect of coagulase-negative staphylococci and *Escherichia coli*, as those bacteria were the most abundant in our groups. The size of our cohort did not allow for further subdivisions in bacterial groups to study the effect of individual bacterial species.

Our results should be interpreted with care due to several limitations including the retrospective nature of the study and the related possibility of bias. A marker of severity of illness would have been a particularly useful variable, but could not be obtained from our data. This study was neither designed nor powered to evaluate the differences in bleeding events and did not take into full account the time relation between the bleeding events and actual occurrence of thrombocytopenia, or other potential factors influencing the risk of bleeding such as hemodynamic instability.

In conclusion, we found that in neonatal sepsis, half of the infants develop thrombocytopenia and 20% develop severe thrombocytopenia. Thrombocytopenia is independently associated with Gram negative (as opposed to Gram positive) sepsis, maternal hypertension and intravascular thrombosis. Severe thrombocytopenia shows an increased association with major hemorrhages and mortality. Both thrombocytopenia and Gram negative sepsis are independently associated with mortality. It is important to take these risks into account in the care of septic neonates. Nevertheless, the exact pathogenesis of thrombocytopenia is still poorly understood and warrants further research. Ideally, a prospective multicentre study would be numerically able to stratify to exact bacteria, rather than differentiate only between Gram negative or positive stain. There is need of well-designed clinical trials regarding platelet transfusion thresholds and the development of a prediction model by which bleeding risk for individual neonates can be estimated.

## Supporting information

S1 File Data on thrombocytopenia and sepsis. SPSS data file containing anonymized data.
(SAV)
